# Evaluating event-based surveillance capacity in Africa: Use of the Africa CDC scorecard, 2022–2023

**DOI:** 10.1016/j.pmedr.2023.102398

**Published:** 2023-09-09

**Authors:** Kyeng Mercy Tetuh, Stephanie J. Salyer, Dativa Aliddeki, Bethelhem Tibebu, Fatma Osman, Franck Chi Amabo, Leocadia Kwagonza Warren, Maryam Ibrahim Buba, Yenew Kebede

**Affiliations:** a Africa Centres for Disease Control and Prevention, Division of Surveillance and Disease Intelligence, Addis Ababa, Ethiopia; b United States Centers for Disease Control and Prevention, Division of Global Health Protection, Atlanta, GA, USA

**Keywords:** Event-based Surveillance, Early warning systems, Capacity scorecard, Evaluation, Africa

## Abstract

**Introduction:**

Event-based surveillance (EBS) is a critical component of Early Warning, Alert and Response (EWAR) capacity needed for outbreak prevention and control. To better understand existing EBS and monitor the progress of capacity-building efforts over time, Africa CDC developed an EBS scorecard as part of a revision to the EBS Framework.

**Methods:**

We distributed the scorecard to African Union (AU) Member States (MSs). Survey responses from the MSs’ human health sector were aggregated, cleaned, and analysed. MS, regional, and continental EBS capacity was assessed.

**Results:**

Between 21 July 2022 and 4 April 2023, a total of 63 respondents representing 49 (89%) of 55 MSs completed the survey. Given Africa CDC’s public health mandate, we acknowledged the importance of One Health collaboration in MSs but focused on and analysed only the human health sector responses. Thirty-four (71%) MSs stated having EBS in place; hotline was the most common type of EBS implemented (76%). Seventeen (50%) MSs reported multisectoral, One Health collaboration as part of EBS implementation. Scorecard outcomes showed a minimal (score of <60%) to average (score between 60–80%) level of EBS capacity in 29 and five (5) MSs respectively.

**Discussion:**

Current EBS capacity levels need to be strengthened in Africa to ensure the continent remains prepared for future public health threats. The Africa CDC EBS scorecard provides a useful way to measure and track this capacity over time. Results can be used to advocate for and target resources for capacity building to foster public health emergency preparedness efforts.

## Highlight

• At least 34 African Union Member States have some form of Event Based Surveillance in place.
• The most common form of EBS in place is hotline and the least implemented is community-based EBS.
• The Africa CDC EBS framework and the IDSR Strategy are the most referenced guideline for EBS in Africa.
• There is no African Union (AU) Member State (MS) yet with optimal capacity for event-based surveillance.
• At 50% of AU MS are using the One-health approach to improve early detection and reporting of health threats.

## 1 Introduction

Africa is home to 22 of the 25 most vulnerable national health systems in the world when considering infectious disease risk factors (Moore et al., 2016). The frequent occurrences of epidemics in low and middle-income countries, including those in Africa, constitutes a significant public health threat to population health and health system strengthening efforts in these countries (Babalola et al., 2022, World Health Organization, 2016, Chackalackal et al., 2021, Worsley-Tonks et al., 2022). Several African Union (AU) Member States (MSs) have made efforts to strengthen their surveillance systems in the last decade - with 44 African countries implementing the World Health Organization’s (WHO) Integrated Disease Surveillance and Response Strategy (IDSR) (Mremi et al., 2021). IDSR was revised in 2019 to include Event-Based Surveillance (EBS) as a critical component of early detection. EBS is the organized collection, monitoring, assessment, and interpretation of primarily unstructured ad hoc information regarding health-related events or risks that may represent an acute risk to human, animal, plant, or environment health (Africa CDC, 2023). EBS has five main steps: detection, triage, verification, risk assessment, and alert for action and response. EBS as part of epidemic intelligence is critical to Early Warning, Alert and Response (EWAR) as it supports the early detection of events of public health importance (World Health Organization, 2022, Implementation of Early Warning and Response with a Focus on Event-Based Surveillance Interim Version, 2014). Epidemic intelligence related activities have proven useful to improving EWAR for outbreaks in Africa (Williams et al., 2021, Warsame et al., 2020). Between 2017 and 2019, the World Health Organization Africa Regional Office reported a total of 415 infectious disease outbreaks that were detected through epidemic intelligence, of which 25% were specifically detected through EBS, mainly media scanning, and 75% through Indicator-Based Surveillance (IBS). Media scanning, the active monitoring of media-related sources (e.g.; web-based, radio, television, newspapers) on a continuing basis to get information about potential health threats, alone was found to initially detect over 50% of outbreaks of diseases of unknown origin, vaccine derived polio, cholera, anthrax, influenza A H1N1, and guinea worm disease (Africa CDC, 2018). EBS, therefore, plays a critical role in EWAR, especially at the country level, highlighting an urgent need to strengthen the EBS capacities in Africa to effectively detect and respond to public health threats.

To help improve EBS capacity in Africa, Africa Centres for Disease Control and Prevention (Africa CDC) developed a framework for EBS in 2018 (Africa CDC, 2018) and has since supported at least 18 (33%) AU MSs with the adaptation of this framework. Specifically, 15 (27%) MSs were supported in establishing functional EBS units (including multisectoral technical working groups), training at least 2,000 public health practitioners from 16 MS, and establishing three communities of practice in the Central, Northern and Southern regions (Mercy et al., 2023). AU MS were supported using a One Health approach to permit the human, animal, and environment health sectors to detect and share information to respond in real time to shared health threats.

There is a global realisation that EBS and EWAR capacity levels should be measured and tracked to help improve the timeliness of detection and response (Frieden et al., 2021, Smolinski et al., 2017, Clara et al., 2020, Joint External Evaluation Tool: International Health Regulations, 2005, Fieldhouse et al., 2022). While there have been efforts to assess EBS implementation and performance at the country (Malik et al., 2022, Merali et al., 2020, Clara et al., 2020, Kuehne et al., 2019) and continental (Williams et al., 2021, Fall et al., 2019) level in Africa, these assessments have mainly been focused on specific EBS types (e.g., hotline, media scanning, facility-based or community-based). Thus, there is limited information on other types of EBS being implemented and what core EBS capacities are in place. To address this, a section on routine monitoring and evaluation, as well as a capacity assessment scorecard was included in the 2023 revision of the Africa CDC EBS framework. The Africa CDC scorecard was specifically developed to understand the current AU MS EBS capacity and help measure progress over time toward the implementation of EBS (Africa CDC, 2023).

We describe the implementation and initial outcomes of this scorecard implementation in AU MS as a way to inform future areas of EBS capacity building on the African continent.

## 2 Methods

As part of the 2023 revision to the Africa CDC EBS framework, the Africa CDC developed an EBS scorecard (Africa CDC, 2023) (see Annex 7). The scorecard was developed through regional consultative workshops with subject matter experts on surveillance from the human, animal, and environment health sectors. The final version was validated by AU MSs actively implementing the Africa CDC EBS framework. This scorecard measured 10 different domains that correspond with the National Public Health Institute (NPHI) capacity scorecard domains and links to the WHO Joint External Evaluation (Joint External Evaluation Tool: International Health Regulations, 2005) (JEE) capacities. These linked JEE indicators are for surveillance (D2.1–2.3), national laboratory system (D1.3), health emergency management (R1.1), points of entry and border health (PoE1), human resources (D3.1, 3.3, 3.4), and legal instruments (P1.1). We distributed a survey version of this scorecard to all 55 AU MSs to evaluate its use and to describe existing EBS capacity in Africa.

For ease of dissemination, the English version of the scorecard listed in the EBS framework was slightly modified and converted into a Google survey format (see supplemental materials). Three additional questions were added to identify what EBS resources were being used, what types of EBS were in place and at what levels within the MS, and lastly, what sectors in the MS were involved with EBS. This survey was then translated into French and Arabic to address the language preference of respondents. The survey was distributed in person to participants of Africa CDC-led regional EBS meetings held in July and August 2022 and by email starting in January 2023 to all MS-level EBS focal points not covered by the regional meetings. Given that some MSs identified multiple EBS focal points to represent the various health sectors, there was a possibility for some MSs to submit more than one survey for their country and representative health sector. Respondents were contacted post-survey where additional clarification or consolidation was needed regarding survey responses. An initial analysis was presented at the Africa CDC EBS Framework launch in March 2023 in Nairobi, Kenya. During this meeting additional MS responses and response clarification was obtained from the Ministry of Health and NPHI surveillance staff in attendance.

All survey responses were combined into a Microsoft Excel spreadsheet and translated back into English for data cleaning and descriptive analysis. Given the public health mandate of Africa CDC, we focused on and analysed only the human health sector responses, but took into consideration the amount of multisectoral, One Health collaboration, where noted. Scores were only calculated for MSs where EBS was in place at the time of the survey. Methods for calculating the domain and overall scores are described in the revised EBS Framework (Africa CDC, 2023). We aggregated and averaged implementation and scorecard data at the AU regional and continental level and provided overall scores by indicator level. Scores were classified as optimal (>80%), average (60–80%) and minimal (<60%).

Email addresses of survey respondents were obtained to allow for follow-up on responses; however, no other identifiable information was taken or used as part of this survey. The US CDC and Africa CDC deemed this a non-research, routine program activity.

## 3 Results

Between 21 July 2022 and 4 April 2023, 63 respondents completed 67 scorecards. These respondents represented 49 (89%) MSs. Respondents represented the human health sector (e.g., ministry of health) in 48 MSs, the animal health sector (e.g., ministry of agriculture) in eight MSs, the environmental health sector (e.g., ministry of environment) in three MSs, and one MS’s research institution (Table 1).

**Table 1:** Sectors of Africa CDC scorecard survey respondents, 21 July 2022 to 4 April 2023.

**AU Region**	**Number of surveys completed**	**Number of respondents**	**Number (%) of MSs represented**	**Number of MS-level health sectors represented**			
				**Human**	**Animal**	**Environment**	Other*
**Central**	18	17	8 (89)	8	4	3	0
**Eastern**	12	12	12 (86)	12	0	0	0
**Northern**	7	7	6 (86)	6	2	0	0
**Southern**	13	12	10 (100)*	9	2	0	0
**Western**	17	15	13 (87)	13	0	0	1
**Total**	**67**	**63**	**49 (89)**	**48**	**8**	**3**	**1**

* Note that while 100% of Member States (MS) in the Southern region had respondents, only 9 of these 10 were from the human health sector, and only these 9 were considered in further analysis and tabulation. “Other” represents a research institution in this instance.

Respondents and MS focal points from 29 (60%) MSs were contacted after the survey submission review for consolidation and alignment; 15 MSs were contacted to resolve multiple surveys that were submitted from the same MS, and 19 for additional clarification on survey responses.

**Fig. 1:**
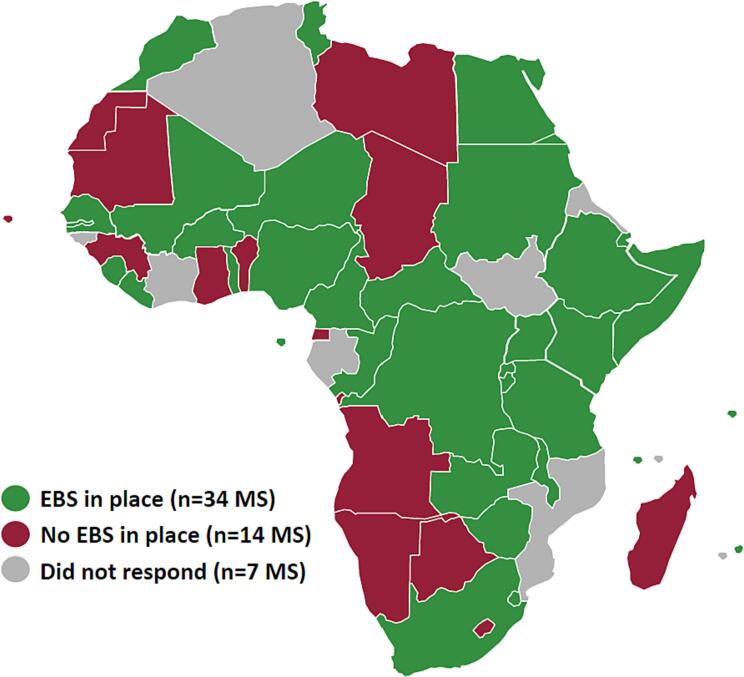
Map of African Union Member States (MS) with at least one type of event-based surveillance in place as of April 2023.

While 49 MSs responded to the survey, we only evaluated the EBS capacity of those MSs where a respondent represented the public health sector, which was 48. From these 48 MSs, 34 (71%) stated having EBS in place at the time of the survey. Per region, the human health sector survey response rate was 89% (n = 8) for the Central region, 86% (n = 12) for the Eastern region, 86% (n = 6) for the Northern region, 90% (n = 9) for the Southern region, and 87% (n = 13) for the Western region (Fig. 1). Of these respondents, 75% (n = 6) of the Central region MSs had EBS in place, with 91% (n = 11) in place in the Eastern, 50% (n = 3) in the Northern, 56% (n = 5) in the Southern, and 69% (n = 9) in the Western regions (See Table 2).

**Table 2:** Event-based surveillance implementation across Africa as stated by Africa CDC event-based surveillance scorecard survey respondents between 21 July 2022 and 4 April 2023.

**Region (number of responding MSs)**	**Number (%) of MSs implementing EBS in Africa**					**Number (%) of Ministries of Health collaborating with other sectors on EBS**
	**Any type of EBS**	**Hotline**	**Media scanning**	**FEBS**	**CEBS**	
**Central** **(8 MS)**	6 (75)	5 (63)	2 (25)	4 (50)	4 (50)	2 (25)
**Eastern** **(12 MS)**	11 (92)	10 (83)	8 (67)	9 (75)	7 (58)	7 (58)
**Northern (6 MS)**	3 (50)	2 (33)	3 (50)	2 (33)	1 (17)	2 (33)
**Southern (9 MS)**	5 (55)	3 (33)	3 (33)	3 (33)	3 (33)	4 (44)
**Western (13 MS)**	9 (69)	6 (46)	8 (62)	3 (23)	3 (23)	2 (15)
**Total** **(48 MS)**	**34 (71)**	**26 (54)**	**24 (50)**	**21 (44)**	**18 (38)**	**17 (35)**

EBS = event-based surveillance; FEBS = Facility-based EBS; CEBS = community-based EBS; MS = Member State. Responses to EBS categories are not mutually exclusive.

For those MSs reporting implementing EBS (34 MSs), the most common form of EBS in place was a hotline (26 MSs; 76%), followed by media scanning (24; 71%), facility-based EBS (FEBS) (21; 62%), and then community-based EBS (CEBS) (18; 53%). Ten (29%) MSs (Cameroon, Egypt, Ethiopia, Kenya, Mali, Nigeria, Sudan, Tanzania, Uganda, and Zambia) stated having all four types of EBS in place. Seventeen (50%) MSs reported the human health sector collaborating with other sectors, primarily with the ministries responsible for animal health (17 MSs; 50%) and environmental health (11; 32%). The most frequently referenced EBS guidance for those implementing EBS included the IDSR technical guidelines 3rd edition (WHO, 2019) (32 MS; 94%) and the Africa CDC EBS framework (Africa CDC, 2018) (20; 59%). Other guidance listed included that developed by the MS (3 MSs; 9%), US CDC (2; 6%), and the 2014 WHO EBS and EWAR (Implementation of Early Warning and Response with a Focus on Event-Based Surveillance Interim Version, 2014) (1; 3%).

When looking at the scorecard results by each indicator in Table 3, in the domains of surveillance and disease intelligence, information systems, legislation, and finance, 50% or more of responses were scored as zero. The remainder of the scorecard domains had higher overall scores, and the domain with the highest capacity was laboratory, with 50% of MSs scoring two, and 18% scoring zero. Within the domain average scores, there is variability of specific indicator scores that make up the average; for example, in the domain of surveillance and disease intelligence, most MSs scored a zero for the indicators, however, 94% of MSs at least scored a one for having a priority list of diseases in place (indicator 1.1).

**Table 3:** Aggregate Africa CDC event-based surveillance scorecard survey results by scorecard indicator as stated by survey respondents between 21 July 2022 and 4 April 2023 for 34 African Union Member States implementing EBS.

**EBS Scorecard Indicator**	Number (%) of MSs reporting the following indicator score level*(n = 34)		
	**0**	**1**	**2**
**1) Surveillance and Disease Intelligence**			
**1.1) Has the National EBS technical working group (TWG) prepared and agreed upon a list of priority events for EBS?**	2 (6)	**21 (62)**	11 (32)
**1.2) 80% or more of events detected by EBS in the last 12 months were detected within 7 days of emergence/event start.**	**20 (59)**	7 (21)	7 (21)
**1.3) 80% or more of events detected through EBS in the last 12 months were notified within 24 h of being verified.**	**20 (59)**	7 (21)	7 (21)
**1.4) 80% or more of signals/events reported through EBS channels in the past 12 months had no missing information.**	**26 (76)**	5 (15)	3 (9)
**1.5) 80% or more of signals in the last 12 months were verified within 24 h of being detected by EBS.**	**20 (59)**	5 (15)	9 (26)
**1.6) 80% or more of events detected through EBS in the last 12 months underwent a risk assessment within 24 h of being verified.**	**28 (82)**	3 (9)	3 (9)
**1.7) 80% or more reports regarding EBS events in the last 12 months were disseminated and shared back to reporting entities.**	**23 (68)**	6 (18)	5 (18)

**2) Information Systems**			
**2.1) Country has an electronic event management system (EMS) to manage (e.g., collect, analyse, and disseminate) EBS data**	12 (35)	**19 (56)**	3 (9)
**2.2) The EMS systematically monitors the performance of EBS.**	**21 (62)**	9 (26)	4 (12)
**2.3) The EMS is inter-operable and interconnected within (lab, IBS, etc.) and with other sectors and countries to support coordinated multisectoral, One Health and cross-border surveillance.**	**27 (79)**	7 (21)	0 (0)

**3) Laboratory Systems & Networks**			
**3.1) Country's laboratory network has the capacity to test for at least 80% of pathogens associated with the priority EBS events.**	6 (18)	11 (32)	**17 (50)**

**4) Preparedness and Response**			
**4.1) 80% of events in the last 12 months have completed an effective initial response within 7 days of notification.**	9 (26)	**13 (38)**	12 (35)
**4.2) 80% or more of staff in the rapid response units in the past 12 months participated in at least one training to improve their EBS response coordination knowledge and skills.**	**24 (71)**	4 (12)	6 (18)

**5) Public Health Research & Institutes**			
**5.1) The EBS programme systematically uses operational research evidence from EBS data to improve the country's early warning and response (EWAR) capacity.**	16 (47)	**17 (50)**	1 (3)

**6) Legislation**			
**6.1) The EBS program has legal authority or a policy in place that authorises the collection, sharing, and use of data collected across multiple sectors to conduct coordinated surveillance.**	**17 (50)**	11 (32)	6 (18)
**6.2) The EBS has legal authority or a policy in place that authorises the collection, sharing, and use of data collected across multiple countries to conduct cross-border surveillance.**	**17 (50)**	12 (35)	5 (15)

**7) Finance**			
**7.1) EBS funding mechanism. Who is currently funding EBS in the country?**	**28 (82)**	2 (6)	4 (12)
**7.2) Is the annual work plan/implementation plan for EBS fully funded for the current year?**	**16 (47)**	18 (53)	0 ()

**8) Workforce**			
**8.1) Does the NPHI/MoH have a surveillance workforce development strategy/plan inclusive of EBS?**	**18 (53)**	11 (32)	5 (15)
**8.2) Are the EBS staff at national level trained on all recommended competencies?**	6 (18)	**21 (62)**	7 (21)
**8.3) The EBS program provided supportive supervision to at least 80% of sub-national reporting entities in the last 12 months to improve data collection and timeliness.**	**26 (76)**	5 (15)	3 (9)

**9) Strategic Plan**			
**9.1) The surveillance programme has a strategic plan inclusive of EBS?**	12 (35)	**11 (32)**	11 (32)
**9.2) Is there an annual work plan/implementation plan for EBS?**	11 (32)	11 (32)	**12 (35)**
**9.3) Is there an EBS monitoring and evaluation plan in place?**	**19 (56)**	11 (32)	4 (12)

**10) Structure**			
**10.1) How is the EBS structured in the country?**	13 (38)	**19 (56)**	2 (6)

* Methods for how to determine each indicator level for scoring is described in detail in the Africa CDC EBS framework (Kuehne et al., 2019). In short, these scores can be interpreted as 0 = no capacity, 1 = some capacity, 2 = optimal capacity.

**Fig. 2:**
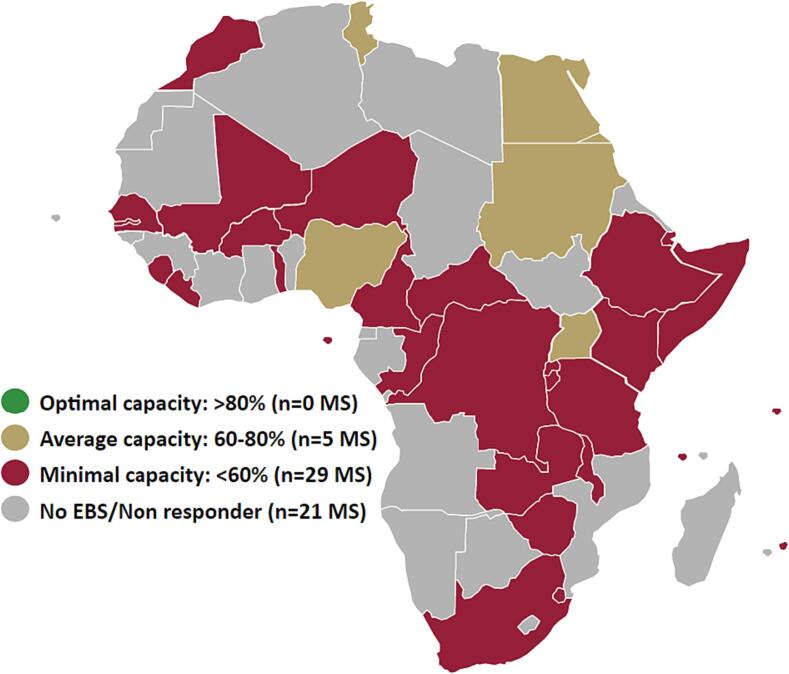
Map of overall Africa CDC EBS scorecard percent scores by African Union Member State (MS) as of April 2023.

Table 4 presents an overall sense of the scores for each domain, and differences in these scores by region. The domain scores across all regions averaged 34%, with laboratory systems and networks, strategic plan, and preparedness and response as the only domains above 35%, at 66%, 44%, and 39%, respectively. The lowest scoring domain was finance at 21%, with the rest ranging between high 20% to low 30% scores. Overall scores were considerably higher in the Northern region at 59%, followed by the Eastern (35%), Southern (29%), Western (29%), and Central (25%) regions. From the ranges shown, there was considerable score variability amongst MSs. At the country level (Fig. 2), 29 (85%) MSs reported minimal EBS capacity, 5 (15%) reported average capacity, and no MS reported having optimal EBS capacity.

**Table 4:** Average continental and regional Africa CDC event-based surveillance scorecard domain and overall percent scores (and country ranges) calculated from survey responses of 34 African Union Member States (MSs) implementing event-based surveillance, collected between 21 July 2022 and 4 April 2023.*

* <60% is considered minimal capacity (red), 60–80% average capacity (orange), and >80% ideal capacity (green) (from Africa CDC Event-Based Surveillance Framework - Annex 7 (Kuehne et al., 2019)).

## 4 Discussion

We noted that the overall EBS capacity on the continent is currently minimal to average at best. The type of EBS being implemented across the continent and regions was variable, with only 10 MSs implementing all four types of EBS. Hotline EBS was the most prevalent type implemented; however, evidence shows that some hotlines have been implemented to support health promotion activities and interventions (Chisholm et al., 2011, Brody et al., 2020) rather than the reporting of public health events to Emergency Operating Centres (EOCs) or epidemic intelligence units. In 2021, Africa CDC received a request for the establishment of EBS in five MSs with a hotline. Following on-site visits to these hotline centres, none had standard operating procedures and operational guidelines on EBS.

For the 71% of MSs implementing media scanning, it is important to understand the biosurveillance tools being used for routine media scanning and if the relevant country-specific sources and categories are being integrated into these tools (e.g., EIOS) to ensure the appropriate level of media granularity is being considered. It is encouraging to see that more than half of MSs implementing EBS are implementing FEBS and CEBS as these are the most optimal in capturing events earlier and closer to the source (Merali et al., 2020, Kuehne et al., 2019, Clara et al., 2018). However, this should continue to be further expanded to ensure that there is country-wide and cross border coverage in addition to taking a multisectoral, One Health approach in detecting signals (Worsley-Tonks et al., 2022, Danquah et al., 2019).

In our analysis, most of the regions had similar scores suggesting equal capacity, but within the regions, there was great variability from MS to MS in their scores. This suggests that for resource allocation, being at least as granular as the MS level will help with proper resource distribution. It also suggests an opportunity where MSs within a region who scored relatively low may be able to receive mentorship from the higher scoring MSs with well-established capacity when ramping up EBS. MS could also benefit from peer-to-peer support during exchange visits and information sharing through established regional EBS communities of practice.

Incorporating a multisectoral, One Health approach into EBS is extremely important given the impact that issues like climate change, food security, and zoonotic disease have on health outcomes (Vora et al., 2023). It is no coincidence that most emerging infectious diseases as well as outbreaks declared public health events of international concern are zoonotic or have resulted from a spillover event (Jones et al., 2008, Vora et al., 2022, Bernstein et al., 2022). Currently, half of the MSs who report implementing EBS are incorporating a multisectoral approach by including health sectors beyond human health. However, this should be further encouraged, especially at the community level where spillover and other One Health related events are most likely to occur (Worsley-Tonks et al., 2022, Vora et al., 2023).

Several limitations of the survey methods were noted, especially around survey deployment and administration, and reporting bias. Given that we had to contact over half of the MSs responding with questions regarding survey response consolidation or to clarify responses that were not apparently logical (e.g., FEBS being conducted at the community level), we understand that deployment by email without extensive sensitization was not optimal for this survey. For some respondents, we were able to clarify certain doubts in their responses during in-person meetings and regional workshops. The exchange regarding the purpose and intent of the scorecard at the regional and framework launch meetings also allowed for respondents to gain a better understanding of what EBS implementation should ideally look like. Most respondents implementing EBS stated referencing the IDSR guidelines; however, the extent to which these technical guidelines were deployed for EBS (e.g.; in developing a multisectoral reporting architecture, in developing signals, or in defining the implementation steps for EBS) could not be assessed. In addition, implementation may solely be considered the addition of one or two signal definitions into the IDSR immediately reportable disease list without ensuring that other supportive components are in place - like a multisectoral technical working group or competent, trained staff who are confident in how to identify and report signals at all levels by all EBS types. The lack of these other supportive components is reflected in the scorecard results that reference these capacities and highlight a sensitization and knowledge gap around EBS that needs to be addressed. Ideal scorecard implementation, may be better achieved through an initial in-person consultation or facilitated review to help eliminate confusion around the indicators. This process could also provide additional feedback on how questions or accompanying instructional materials could be further clarified to improve survey comprehension and responses for future remote deployments. Subsequent assessments could then be conducted by national staff trained on EBS methodology.

To assist countries with improving EBS capacity, the revised Africa CDC EBS framework (Africa CDC, 2023) outlines key considerations for the establishment or strengthening of functional EBS systems. These include establishing national guidelines for the type(s) of EBS being implemented and incorporating a multisectoral, One Health approach in the establishment of EBS coordination groups to ensure all relevant sources and stakeholders are identified and engaged in detecting, reporting, and responding to identified health threats. Countries are recommended to establish a clear reporting architecture and methodology of reporting signals and events across sectors and different administrative levels for prompt action. Countries are also urged to build EBS workforce and foster peer to peer mentorship and conduct supportive supervision for effective implementation of EBS. Other global guidelines like WHO’s Early Warning Alert and Response in Emergencies (World Health Organization, 2022) are also available for reference and provide a systematic approach in the establishment and utilisation of EBS and IBS systems for EWAR during emergencies.

## 5 Conclusion

Event-based surveillance remains vital to EWAR. Current EBS capacity levels need to be strengthened in Africa to ensure the continent remains prepared for future public health threats. The Africa CDC EBS Scorecard provides a useful way to measure and track this capacity over time. Results can be used to advocate for and target resources to the appropriate EBS capacity domains that need strengthening. The scorecard remains a valuable tool for assessing and tracking capacity in AU MSs; however, we suggest that individuals trained on EBS should lead the assessment process.

## Research in context

**Evidence before this study:** The concept of EBS, even though critical in improving the early detection of public health threats, has not been well understood amongst public health workers, including disease surveillance officers. This poor understanding translates into EBS implementation gaps across the continent.
For instance, in some African countries, the absence of guidelines and standard operating procedures to guide the operationalization of EBS within the existing surveillance system was identified as a critical gap in scaling up EBS nationwide. In addition, the need for establishing a reporting architecture across administrative levels and sectors has been considered critical in improving EBS performance.
There have been several studies aimed at assessing the implementation of EBS in countries and assessing gaps in EBS performance on the African continent. These assessments have mainly been focused on specific EBS types or core components of EBS implementation. There is limited information on assessments conducted for all EBS types (hotline, media scanning, facility, and community) and core capacities (e.g., multisectoral One Health, event management systems, trained workforce, etc.).
**Added value of this study:** This study provides a comprehensive picture of the EBS capacity on the African continent and provides a framework assessment tool (scorecard) for the continuous evaluation and tracking of EBS capacity within African Union Member States.
**Implications of all the available evidence:** Given the exacerbation of the factors that lead to disease emergence and re-emergence (e.g., population growth and movement, urbanisation, land-use change, climate change, travel, and trade), countries need to continue to assess and strengthen their early warning and response systems to be able to rapidly detect, report and respond to health risks. Assessments like these can help identify key areas that need to be strengthened on a continental, regional, and national level.

## Contributors

KM designed and coordinated the study. FO, DA, BT, LKW, MIB, and FCA acquired the data. KM and SJS did statistical analysis and wrote the report. All authors provided critical conceptual input, analysed, and interpreted data, and critically revised the report. KM and SJS verified the data. All authors had full access to all the data in the study and had final responsibility for the decision to submit for publication.

## Data sharing

To gain access to a complete export of this dataset, please contact AfricaCDCEBS@africa-union.org to sign a data access agreement.

## Funding

None.

## CRediT authorship contribution statement

**Kyeng Mercy Tetuh:** Conceptualization, Formal analysis, Methodology, Validation, Visualization, Writing – original draft, Writing – review & editing. **Stephanie J. Salyer:** Methodology, Visualization, Writing – original draft, Writing – review & editing. **Dativa Aliddeki:** Methodology, Writing – review & editing. **Bethelhem Tibebu:** Methodology, Writing – review & editing. **Fatma Osman:** Methodology, Writing – review & editing. **Franck Chi Amabo:** Methodology, Writing – review & editing. **Leocadia Kwagonza Warren:** Methodology, Writing – review & editing. **Maryam Ibrahim Buba:** Methodology, Writing – review & editing. **Yenew Kebede:** Methodology, Writing – review & editing.

## Declaration of Competing Interest

The authors declare that they have no known competing financial interests or personal relationships that could have appeared to influence the work reported in this paper.

## Data Availability

Data will be made available on request.
