# Supplementary material for: Evaluating event-based surveillance capacity in Africa: Use of the Africa CDC scorecard, 2022–2023
Source: Prev Med Rep. 2023 Sep 9;36:102398. doi: 10.1016/j.pmedr.2023.102398 (PMC10502352; doi:10.1016/j.pmedr.2023.102398)
Supplement: Supplementary data 1 [file mmc1.docx]

**Supplemental Materials**

**Event-based Surveillance Capacity Survey**

Email: ____________________________

What country are you representing? ____________________________

What sector/ministry are you representing? ____________________________

**Surveillance and Disease Intelligence**

1) Has the National EBS TWG prepared and agreed upon a list of priority events for EBS? **Documentation to reference:** List of priority events (e.g., IDSR list), Terms of Reference of national EBS TWG

*(Note - this relates to scorecard question 1.1*)

a. No EBS is in place for priority events (0)

b. EBS established for at least one priority event (1)

c. EBS is functional for all priority events AND signal definitions are in place at all levels (e.g., national, facility, and community) to improve early warning and response (2)

2) If EBS is established what type of EBS is in place at what level?

|  | No EBS in place | Hotline | Media Scanning | Facility-Based | Community-Based |
| --- | --- | --- | --- | --- | --- |
| No EBS in place | o | o | o | o | o |
| National Level | o | o | o | o | o |
| Intermediate Level (e.g., district) | o | o | o | o | o |
| Facility Level | o | o | o | o | o |
| Community Level | o | o | o | o | o |

3) What sectors are involved in EBS implementation?

|  | No EBS in place | Hotline | Media Scanning | Facility-Based | Community-Based |
| --- | --- | --- | --- | --- | --- |
| No EBS in place | o | o | o | o | o |
| Public Health (e.g., Ministry of Health) | o | o | o | o | o |
| Domestic Animal Healthy (e.g., Ministry of Agriculture) | o | o | o | o | o |
| Environmental health (e.g., Ministry of Environment; Ministry of Water and Sanitation) | o | o | o | o | o |
| Plant health (e.g., Ministry of Agriculture; Ministry of Environment) | o | o | o | o | o |
| Wildlife health (e.g., Ministry of Agriculture; Ministry of Environment; Ministry of Tourism) | o | o | o | o | o |

4) What guidance documents have you used to help guide the implementation of your EBS?

a. Africa CDC EBS framework

b. WHO IDSR Strategy

c. Other: __________________

5) What percentage of epidemics (above epidemic/alert thresholds) detected through the routine indicator-based surveillance (e.g., IDSR) in the last 12 months were reported to the EBS as signals within 24 hours of detection?

**Documentation to reference:** Trend analysis of selected conditions, M&E framework or data

a. 0-50% (0)

b. 51-79% (1)

c. ≥ 80% (2)

6) What percentage of events detected by EBS in the last 12 months were detected within 7 days of emergence/event start*?

***Date of emergence/event start**: typically, the date of symptom onset in the index case, but typically the earliest date associated with the start of an event (e.g., symptom-onset date of first reported case; first visit to health care facility; date of suspected primary case; most likely exposure date; first exposure date; latest exposure date; outbreak start date; date of death of first reported case; first report of the outbreak)

**Documentation to reference:** M&E framework or data, 717 metrics

*(Note - this relates to scorecard question 1.2)*

a. 0-50% (0)

b. 51-79% (1)

c. ≥ 80% (2)

7) What percentage of events detected through EBS in the last 12 months were notified within 24 hours of being verified?

**Documentation to reference:** M&E framework or data

*(Note - this relates to scorecard question 1.3)*

a. 0-50% (0)

b. 51-79% (1)

c. ≥ 80% (2)

8) What percentage of signals/events reported through EBS channels in the past 12 months had no missing information? Key variables to look at include: date of event start, detection, verification, risk assessment, response

**Documentation to reference:** Event management system (e.g., DHIS-2, SORMAS, EWARS)

*(Note - this relates to scorecard question 1.4)*

a. 0-50% (0)

b. 51-79% (1)

c. ≥ 80% (2)

9) What percentage of signals in the last 12 months were verified within 24 hours of being detected by EBS?

**Documentation to reference:** M&E framework or data

*(Note - this relates to scorecard question 1.5)*

a. 0-50% (0)

b. 51-79% (1)

c. ≥ 80% (2)

10) What percentage of events detected through EBS in the last 12 months underwent a risk assessment within 24 hours of being verified?

**Documentation to reference:** M&E framework or data

*(Note - this relates to scorecard question 1.6)*

a. 0-50% (0)

b. 51-79% (1)

c. ≥ 80% (2)

11) What percentage of reports regarding EBS events were disseminated and shared back with reporting entities in the last 12 months?

**Documentation to reference:** reports include situation reports and spot reports

*(Note - this relates to scorecard question 1.7)*

a. 0-50% (0)

b. 51-79% (1)

c. ≥ 80% (2)

**Information Systems**

12) Country has an electronic event management system (EMS) to manage (collect, analyse, and disseminate) EBS data.

**Documentation to reference:** EMS use guidelines/SOPs.

*(Note - this relates to scorecard question 2.1)*

a. There is no EMS in place (0)

b. An EMS is in development or only partially in use (1)

c. An EMS is in place and managing data for all levels and types of EBS in place within the country (2)

13) Does the EMS systematically monitor the performance of EBS? Performance can be monitored by assessing completeness and accuracy of data entered into the system. This also includes the ability to integrate M&E indicators associated with EBS performance like timeliness indicators and key dates (e.g., date of event start, detection, verification, risk assessment, response).

**Documentation to reference:** EMS SOP, M&E plan, key performance indicators, 717 plan

*(Note - this relates to scorecard question 2.2)*

a. The EMS does not monitor the performance of EBS (0)

b. The EMS monitors the performance of EBS ad hoc (1)

c. The EMS monitors the performance of EBS systematically and continuously (2)

14) Is the EMS inter-operable and interconnected within (e.g., lab, IBS) and between different sectors (e.g., human, animal, environment) and countries to support coordinated multisectoral, One Health and cross-border EBS.

**Documentation to reference:** EMS use guidelines/SOPs

*(Note - this relates to scorecard question 2.3)*

a. The EMS is not interoperable or interconnected (0)

b. The EMS is partially connected within the public health and with other sectors (1)

c. The EMS is fully connected within the public health sector and countries to support coordinated and cross-border surveillance (2)

**Laboratory Systems and Networks**

15) Does your country's laboratory network have the capacity to test for at least 80% of pathogens associated with the priority EBS events?

**Documentation to reference:** Laboratory data; M&E framework or data

*(Note - this relates to scorecard question 3.1)*

a. 0-50% (0)

b. 51-79% (1)

c. ≥ 80% (2)

**Preparedness and Response**

16) What percent of events in the last 12 months completed an effective initial response within 7 days of notification? **Documentation to reference:** M&E and 717 M&E plan, 717 metrics for "effective initial response" see 717 supplemental materials. Date of effective initial response: date when all of the following 7 actions are completed: initiate investigation/response, epidemiological investigation, laboratory confirmation, initiate case management, initiate countermeasures, initiate communications and community engagement, establish response coordination mechanism (see 717 for more details) *(Note - this relates to scorecard question 4.1)*

a. NPHI/MOH responded to 0-50% of notifications within 7 days (0)

b. NPHI/MOH responded to 51 - 79% of notifications within 7 days (1)

c. NPHI/MOH responded to ≥ 80% of notifications within 7 days (2)

17) What percentage of rapid response unit staff in the last 12 months participated in at least one training to improve their EBS response coordination knowledge and skills?

**Documentation to reference:** Training reports

*(Note - this relates to scorecard question 4.2)*

a. 0-50% (0)

b. 51-79% (1)

c. ≥ 80% (2)

**Research**

18) The EBS programme systematically uses operational research evidence from EBS data to improve the country's early warning and response (EWAR) capacity.

**Documentation to reference:** EBS data informed reports and publications

*(Note - this relates to scorecard question 5.1)*

a. Operational research evidence is not used (0)

b. Operational research evidence is used but not systematic (1)

c. Operational research evidence is systematically used to improve EWAR capacity (2)

**Legislation**

19) The EBS program has legal authority or a policy in place that authorises the collection, sharing, and use of data collected across multiple sectors to conduct coordinated surveillance.

**Documentation to reference:** Multisectoral data sharing policy/MoU

*(Note - this relates to scorecard question 6.1)*

a. No multisectoral legal authority or policy is in place (0)

b. Legal authority or a policy is in place between at least two sectors for coordinated surveillance (1)

c. Legal authority or policy is in place between all relevant coordinated surveillance stakeholders (2)

20) The EBS has legal authority or a policy in place that authorises the collection, sharing, and use of data collected across multiple countries to conduct cross-border surveillance.

**Documentation to reference:** Cross-border surveillance data sharing policy/MoU

*(Note - this relates to scorecard question 6.2)*

a. No multi-country cross-border legal authority or policy is in place (0)

b. Legal authority or a policy is in place between at least two countries for cross-border surveillance (1)

c. Legal authority or policy is in place between all neighbouring countries for effective cross-border surveillance (2)

**Finance**

21) EBS funding mechanism - Who is currently funding EBS in the country?

**Documentation to reference:** Annual work plan specifying source of funding

*(Note - this relates to scorecard question 7.1)*

a. 0-50% of the funding is provided by the country (0)

b. 51-79% of the funding is provided by the country (1)

c. ≥ 80% of the funding is provided by the country (2)

22) Is the annual implementation plan for EBS fully funded?

**Documentation to reference:** Annual work plan specifying source of funding

*(Note - this relates to scorecard question 7.2)*

a. The EBS plan is not funded (0)

b. The plan is partially funded (1)

c. The plan is fully funded (2)

**Workforce**

23) Does the NPHI/MOH have a surveillance workforce development strategy/plan inclusive of EBS?

**Documentation to reference:** Surveillance workforce development strategy/plan

*(Note - this relates to scorecard question 8.1)*

a. The EBS programme does not have a workforce development strategy (0)

b. The EBS programme is in the process of developing a workforce development strategy/plan (1)

c. The EBS programme has a workforce development strategy/plan (2)

24) Is the national team trained on EBS for all recommended competencies?

**National level training competencies include:** 1) types of EBS (e.g., media, hotline, facility, community); 2) M&E (knowledge of indicators); 3) innovation (e.g., EMS, analytics)

**Documentation to reference:** EBS training records

*(Note - this relates to scorecard question 8.2)*

a. No training provided on EBS (0)

b. Training provided in EBS on some but not all competencies (1)

c. All EBS staff at the national level are competent in all training competencies (2)

25) What percentage of sub-national reporting entities were provided supportive supervision by the EBS program in the last 12 months to improve data collection and timeliness?

**Documentation to reference:** Supportive supervision reports

*(Note - this relates to scorecard question 8.3)*

a. 0-50% (0)

b. 51-79% (1)

c. ≥ 80% (2)

**Plans (e.g., strategic, annual work plans, M&E)**

26) The surveillance programme has a strategic plan inclusive of EBS?

**Documentation to reference:** Surveillance program strategic plan

*(Note - this relates to scorecard question 9.1)*

a. There is no strategic plan (0)

b. The strategic plan is in development (1)

c. The plan has been developed and is in place (2)

27) Is there an annual workplan/implementation plan?

**Documentation to reference:** EBS annual work plan

*(Note - this relates to scorecard question 9.2)*

a. There is no annual workplan/implementation in place (0)

b. The plan is in development (1)

c. There is well-developed annual work plan/implementation plan (2)

28) Is there an EBS monitoring and evaluation plan in place?

**Documentation to reference:** EBS monitoring and evaluation plan, IDSR guidelines

*(Note - this relates to scorecard question 9.3)*

a. No M&E plan established (0)

b. There is a plan that is reviewed on ad-hoc basis (1)

c. There is plan that is tracked/monitored regularly (2)

**Structure**

29) How is the EBS structured in the country?

**Multilevel:** EBS is implemented at the national, intermediate, facility, community levels

**Multisectoral:** EBS is inclusive of the One Health approach and includes linkages to all relevant sectors (e.g., human, animal, environment)

**Cross-border:** EBS includes linkages with neighbouring countries, taking a regional approach **Documentation to reference:** EBS SOPs, work plans/implementation plans.

*(Note - this relates to scorecard question 10.1)*

a. EBS doesn't have multi-level, multisectoral, or cross-border linkages (0)

b. EBS has initiated multi-level, multisectoral, and cross-border linkages (1)

c. EBS has well-established multi-level, multisectoral, and cross-border linkages (2)
